# Low density lipoprotein from patients with Type 2 diabetes increases expression of monocyte matrix metalloproteinase and ADAM metalloproteinase genes

**DOI:** 10.1186/1475-2840-6-21

**Published:** 2007-08-22

**Authors:** Joanna R Worley, David A Hughes, Nicoletta Dozio, Jelena Gavrilovic, Mike J Sampson

**Affiliations:** 1 School of Biological Sciences, University of East Anglia, Norwich, NR4 7TJ, UK; 2 Bertram Diabetes Research Department, Norfolk and Norwich University Hospital, Norwich, NR4 7UY, UK; 3 Institute of Food Research, Norwich, NR4 7UA, UK

## Abstract

**Aims:**

Type 2 diabetes is characterised by increased plasma concentrations of pro-inflammatory cytokines [such as tumour necrosis factor – alpha; TNF-α] and soluble forms of adhesion molecules involved in leukocyte – endothelial interactions. These molecules are synthesised as transmembrane proteins and the plasma soluble forms are generated by ectodomain cleavage from the cell surface by members of the ADAM [adisintegrin and metalloproteinase] proteinase family. We hypothesised that plasma low density lipoprotein [LDL] from subjects with Type 2 diabetes would influence *in vitro *monocytic ADAM and matrix metalloproteinase [MMP] gene expression differently compared to control LDL.

**Methods:**

We examined relative mRNA expression by real time PCR in a monocytic cell line [THP-1] cultured for 4, 8 and 24 hrs with human plasma LDL derived from subjects with [n = 5] or without [n = 4] Type 2 diabetes. Gene expression for MMP-1 and 9, and ADAM – 8, 15, 17 and 28 was studied.

**Results:**

Type 2 diabetes LDL significantly increased gene expression of MMP – 1 [p < 0.01] MMP – 9 [p < 0.001], and ADAM 17 [p < 0.05], – 28 [p < 0.01] and – 15 [p < 0.01] compared to control LDL. Type 2 diabetes LDL had disparate effects on inhibitors of MMP.

**Conclusion:**

These data suggest that Type 2 diabetes LDL could lead to increased adhesion molecule and TNF alpha cell surface shedding, and vascular plaque instability, by promoting increased expression of ADAM and MMP genes.

## Background

Type 2 diabetes is characterised by elevated circulating levels of pro – inflammatory cytokines such as Tumour Necrosis Factor alpha [TNF-α] and the soluble forms of adhesion molecules involved in leukocyte – endothelial cell interactions, such as intercellular adhesion molecule-1 [ICAM-1], vascular cell adhesion molecule-1 [VCAM-1] and E-selectin. [1]. These abnormalities may be atherogenic, and overexpression and release of TNF-α may have a role in the development of insulin resistance and Type 2 diabetes [2].

Cell surface adhesion molecules and TNF-α are synthesised as transmembrane proteins, and the plasma soluble forms are generated by ectodomain cleavage from the cell surface. Ectodomain shedding of cell membrane forms is mediated by certain members of the ADAM [**a d**isintegrin **a**nd **m**etalloproteinase] proteinase family [3]. The catalytic domain of ADAMs share homology with the matrix metalloproteinases [MMP], which have a role in vascular plaque stability [4]. ADAM17 is involved in shedding vascular cell adhesion molecule 1 [VCAM-1] [3], L-selectin [3] and other cell membrane proteins including TNF-α and its receptor [3]. The ADAM proteinases also have a role in cell: cell and/or cell: matrix interactions [3]. The elevated plasma levels of soluble TNF-α and some adhesion molecules in Type 2 diabetes could imply increased activity or expression of ADAMs in these observations.

Plasma LDL from people with Type 2 diabetes is structurally and biochemically different, and commonly minimally oxidatively modified [5]. However, it is unknown how modified LDL derived from people with Type 2 diabetes influences MMP or ADAM gene expression *in vitro *or *in vivo*. We have previously shown in monocytic cells that ADAM mRNA expression can be regulated by PPAR-gamma agonists [6], which is relevant as components of oxidatively modified LDL can be agonists of PPAR-gamma. We hypothesised that plasma LDL from people withType 2 diabetes would influence *in vitro *monocytic ADAM and MMP gene expression differently compared to control LDL.

## Methods

### Subjects [Table 1]

**Table 1:** Baseline data for Type 2 diabetes and control plasma LDL donors

	**TYPE 2 DIABETES**	**CONTROLS**	**p**
**Number**	5	4	
**Age [yrs]**	63.6 [0.93]	53.0 [3.08]	0.02
**Known Diabetes duration [yrs]**	7.8 [0.66]	-	
**M:F**	02:03	02:02	
**Body Mass Index [kg/m^2^]**	30.2 [1.66]	25.5 [0.5]	ns
**Waist – hip ratio**	0.88 [0.02]	0.78 [0.05]	ns
**Diabetes treatment**		-	
Diet	1		
Sulphonylurea	2		
Metformin	1		
Sulphonylurea/metformin	1		
**HbA1c [%]**	6.82 [0.56]	5.10 [0.10]	ns
**Total cholesterol [mmol/l]**	6.68 [0.55]	6.33 [0.70]	ns
**LDL cholesterol [mmol/l]**	4.30 [0.59]	4.4 [0.67]	ns
**Triglycerides [mmol/l]**	2.56 [0.31]	1.39 [0.08]	0.036
**HDL cholesterol [mmol/l]**	1.22 [0.08]	1.32 [0.05]	ns

Data shown as mean and [standard error of mean] ns = not significant.

After Ethical Committee approval and written informed consent, we obtained fasting plasma LDL from subjects with Type 2 diabetes [n = 5] or controls without diabetes [n = 4]. All donors were Caucasian, non-smokers between 45 and 70 years old. Type 2 diabetes was defined as diagnosis after the age of 40 years, no history of ketosis and with stable glycaemic control on diet or oral hypoglycaemics. Patients were excluded if they had hypertension, clinically expressed coronary artery disease, were receiving hormone replacement therapy, aspirin, HMG CoA reductase ['statin'] or insulin therapy. Patients with macroproteinuria were excluded. All controls had fasting plasma glucose below 6.1 mmol/l.

### LDL isolation and characterisation

LDL were isolated from fasted human plasma, containing 1 mg/ml EDTA [Na_2_] and 0.6% [w/v] sucrose, stored at -70°C, using a short-run-ultracentrifugation method based on non-equilibrium density gradient ultracentrifugation. Oxidized LDL in each LDL was determined using the Mercodia Oxidized LDL ELISA kit which measures the aldehyde modification of apolipoprotein B lysine residues

### Cell culture

THP-1 cells were maintained in RPMI 1640 [Life Technologies, UK] supplemented with 5 mM L-glutamine 100 U/ml penicillin and streptomycin and 10% foetal calf serum, at 37°C and 5% CO_2_. For experiments, cells were cultured in serum free RPMI alone, or containing 25, 50 or 100 microg/ml Type 2 diabetes LDL and control LDL in triplicate, for 4, 8 or 24 hours as indicated.

### RNA isolation and Real Time PCR analysis

Total RNA was isolated using the RNA-Bee™ [Biogenesis] according to the manufacturer's instructions. RNA [1 microg] was reverse transcribed in a 20 microl reaction using SuperScript™ II reverse transcriptase [Invitrogen] according to the manufacturer's instructions. Relative mRNA expression [compared to 18S RNA] of ADAM8, 15, 17 and 28, MMP-1, -9 and 14 and TIMP-1, -3 and 4 were measured by Taqman real time PCR [Perkin Elmer] as described previously [6].

### Experimental plan

The choice of genes for MMP, ADAM and metalloproteinase inhibitors was based on our previous studies. [6].

### Statistical analysis

Expression of mRNA for the gene of interest in each RNA sample was calculated as a ratio with 18S RNA. Each biological triplicate was compared to mRNA expression by untreated cells at the appropriate time point. All data was assumed to be non normally distributed. Type 2 diabetes [n = 5] and control [n = 4] values, all in triplicate, at each concentration and time point were compared using Mann-Whitney Rank Sum Test. LDL treatments at each time point were compared to untreated controls using Kruskal-Wallis test, with Dunn's Multiple comparison test.

## Results

Table 2 shows MMP, ADAM and TIMP mRNA expression of cells treated with Type 2 diabetes LDL or control LDL compared to the mRNA expression in untreated cells at each time point.

**Table 2:** mRNA expression for ADAM and MMP genes in response to increasing human LDL concentrations (25,50 and 100 μg/ml from control or Type 2 diabetes donors) after 4,8 and 24 hours culture

	**LDL concentration (μg/ml)**					
	
	***25***		***50***		***100***	
	C	T2DM	**C**	**T2DM**	**C**	**T2DM**
**MMP – 1**						
4 hrs	0.48 (0.09)	0.97 (0.21)	0.41 (0.14)	0.66 (0.11) *	0.29 (0.06)	0.62 (0.09) **
8 hrs	0.69 (0.08)	0.63 (0.07)	0.59 (0.05)	0.53 (0.08)	0.53 (0.07)	0.56 (0.05)
24 hrs	-	-	-	-	-	-
**MMP – 9**						
4 hrs	0.98 (0.09)	1.36 (0.10) *	1.02 (0.15)	1.55 (0.13) *	1.01 (0.19)	1.98 (0.2) **
8 hrs	0.84 (0.05)	0.89 (0.07)	0.76 (0.05)	0.94 (0.05) *	0.73 (0.10)	0.94 (0.07) **
24 hrs	0.89 (0.06)	1.09 (0.06)	0.63 (0.04)	1.0 (0.07) ***	0.67 (0.09)	1.03 (0.08) **
**TIMP – 1**						
4 hrs	0.73 (0.04)	0.88 (0.05)	0.74 (0.04)	0.86 (0.04)	0.73 (0.07)	0.90 (0.05) *
8 hrs	0.94 (0.06)	0.87 (0.04)	0.90 (0.05)	0.86 (0.03)	0.81 (0.04)	0.81 (0.04)
24 hrs	1.09 (0.06)	1.14 (0.07)	0.94 (0.06)	1.08 (0.05)	1.1 (0.10)	1.16 (0.06)
**TIMP – 3**						
4 hrs	0.87 (0.04)	0.84 (0.04)	0.85 (0.03)	0.73 (0.04) **	0.79 (0.02)	0.70 (0.04)
8 hrs	0.95 (0.04)	0.90 (0.06)	0.85 (0.04)	0.84 (0.04)	0.75 (0.04)	0.68 (0.040
24 hrs	0.99 (0.06)	0.87 (0.04)	1.01 (0.07)	0.84 (0.04) **	0.96 (0.05)	0.86 (0.03)
**ADAM – 28**						
4 hrs	0.76 (0.05)	1.07 (0.05)	0.83 (0.11)	1.07 (0.09)	0.78 (0.1)	1.23 (0.06) **
8 hrs	0.84 (0.06)	0.81 (0.04)	0.87 (0.07)	0.83 (0.05)	0.72 (0.05)	0.75 (0.06)
24 hrs	0.97 (0.07)	1.10 (0.07)	0.85 (0.05)	1.15 (0.09) *	0.95 0.11	1.06 (0.06)
**ADAM – 17**						
4 hrs	0.93 (0.10)	1.19 (0.08)	0.81 (0.09)	1.13 (0.08) *	0.84 (0.12)	1.11 (0.06) *
8 hrs	0.94 (0.04)	1.03 (0.05)	0.93 (0.06)	0.86 (0.04)	0.79 (0.05)	0.78 (0.04)
24 hrs	1.08 (0.09)	1.15 (0.07)	0.92 (0.06)	1.13 (0.07)	1.14 (0.13)	1.17 (0.60)
**ADAM – 15**						
4 hrs	0.91 (0.05)	1.07 (0.10)	0.96 (0.09)	1.18 (0.08)	0.91 (0.09)	1.21 (0.06) **
8 hrs	0.99 (0.4)	1.01 (0.06)	1.08 (0.07)	0.95 (0.04)	0.86 (0.04)	0.87 (0.05)
24 hrs	0.87 (0.03)	0.92 (0.07)	0.86 (0.05)	0.87 (0.05)	0.94 (0.08)	0.99 (0.06)

* p < 0.05 ** p < 0.01 *** p < 0.001 for Type 2 diabetes (T2DM) vs control (C) LDL treated cells mRNA expression at each matched LDL concentration and time. Data shown as a mean (SE)

### Monocytic ADAM gene expression [Table 2]

ADAM gene expression in Type 2 diabetes LDL treated cells was significantly higher compared to control LDL treated cells for a] ADAM 28 after 4 and 24 hours at higher LDL concentrations b] ADAM17 after 4 hours at higher LDL concentrations c] ADAM 15 at 4 hours at higher LDL concentrations.

### Monocytic MMP gene expression [Table 2]

MMP expression in Type 2 diabetes LDL treated cells was significantly higher compared to control LDL treated cells for a] MMP-1 after 4 and 8 hours at all LDL concentrations, and b] MMP – 9 at 4, 8 and 24 hours at most LDL concentrations.

### Monocytic TIMP gene expression [Table 2]

TIMP-1 expression in Type 2 diabetes LDL treated cells was significantly higher compared to control LDL treated cells for at 4 hours at high LDL concentrations. TIMP-3 expression in Type 2 diabetesLDL treated cells was significantly lower compared to control LDL treated cells after 4 and 24 hours at higher LDL concentrations.

### Oxidized LDL

No differences in electrophoretic mobility or oxidized LDL content were detected between the control and Type 2 diabetes [Control LDL, 51.44 U/l ± SEM 6.67; Type 2 diabetes LDL, 39.98 U/l ± SEM 4.694. P = 0.225].

## Discussion

We have shown that plasma LDL from subjects with Type 2 diabetes differs from control LDL in its effects on the pattern of expression of MMP, ADAM and TIMP genes in THP-1 monocytic cells. *In vitro*, Type 2 diabetes LDL promoted the expression of the MMP-1, MMP-9, ADAM28, ADAM17 and ADAM15 genes compared to control LDL. This suggests the lipoprotein environment of circulating and sub-endothelial monocytes in Type 2 diabetes could promote altered shedding of membrane-bound cytokines, receptors and adhesion molecules and alter cell-cell interactions.

Ectodomain shedding by ADAM proteinases is highly relevant in view of the well described but unexplained increase in circulating soluble adhesion molecule concentrations and TNF-α in Type 2 diabetes [1]. The mechanism [s] underlying this consistent observation are unknown and it is not fully understood how ectodomain-shedding is regulated *in vivo*. The present data is consistent with the idea that Type 2 diabetes LDL could increase ADAM17 activity in THP-1 monocytes, and their ability to shed TNF-α and VCAM-1 [7]. Type 2 diabetes LDL also induced a significant increase in ADAM28 gene expression. ADAM28 associates with integrin alpha 4 beta 1, the receptor for endothelial VCAM-1 and CS-1 fibronectin, and integrin alpha 4 beta 7, which binds VCAM-1, MadCAM-1 and fibronectin. This suggests that ADAM28 could be involved in regulating monocyte adhesion to the endothelium.

Alterations in monocyte MMP expression induced by LDL found in Type 2 diabetes LDL, [rather than artificially oxidised LDL] has not been investigated before. These data indicates a significant increase in MMP-1 and MMP-9 expression induced by Type 2 diabetes LDL compared to control LDL. In atheromatous plaque, increased expression of MMP-1 and MMP-9 may decrease vascular plaque stability and risk of plaque rupture [4]. If these observations are a reflection of in vivo interactions between LDL and sub endothelial monocyte – macrophages, this could promote increase vascular plaque instability in Type 2 diabetes.

Subtle differences in the compositions of control and Type 2 diabetes LDL populations may be responsible for the differences in gene expression in this paper as there was no difference in the amount of apolipoprotein B oxidised epitopes. A recent study comparing markers of LDL oxidation found that LDL-ketocholesterol and plasma-ox-apoB were significantly higher in Type 2 diabetes compared to control LDL, but found no difference in levels of lyso-phosphatidylcholine [5]. Others have shown both palmitoyl and stearoyl-lyso-phosphatidylcholine to be increased in Type 2 diabetes LDL [8].

One weakness of the present study is the age difference between groups [Table 1]. We have found no relationship between age variability between ages of 40 and 60 and oxLDL concentrations in Type 2 diabetes or controls [9]. As far as we aware there are no data indicating significant change in LDL components across the narrow age difference in this study. In addition, we should emphasise the small group sizes in this study, the significant basline differences in baseline plasma triglycerides, and the need to repeat these observations in larger populations.

Despite these limitations, these data suggests that LDL from subjects with Type 2 diabetes promotes increased monocyte mRNA expression of MMP-1, MMP-9, and ADAM proteinases responsible for inflammatory cytokine and adhesion molecule shedding. These data suggest a pathway between modification of LDL in Type 2 diabetes increased MMP expression and ecto-domain cleavage of adhesion molecules and TNF-α.

## Abbreviations

TNF-α: Tumour Necrosis Factor alpha.

ICAM-1: intercellular adhesion molecule-1.

VCAM-1: vascular cell adhesion molecule-1.

ADAM: a disintegrin and metalloproteinase.

MMP: matrix metalloproteinase.
